# β-Catenin Directly Sequesters Adipocytic and Insulin Sensitizing Activities but Not Osteoblastic Activity of PPARγ2 in Marrow Mesenchymal Stem Cells

**DOI:** 10.1371/journal.pone.0051746

**Published:** 2012-12-18

**Authors:** Sima Rahman, Piotr J. Czernik, Yalin Lu, Beata Lecka-Czernik

**Affiliations:** 1 Department of Orthopaedic Surgery, University of Toledo College of Medicine, Toledo, Ohio, United States of America; 2 Department of Physiology and Pharmacology, University of Toledo College of Medicine, Toledo, Ohio, United States of America; 3 Center for Diabetes and Endocrine Research, University of Toledo College of Medicine, Toledo, Ohio, United States of America; Georgia Health Sciences University, United States of America

## Abstract

Lineage allocation of the marrow mesenchymal stem cells (MSCs) to osteoblasts and adipocytes is dependent on both Wnt signaling and PPARγ2 activity. Activation of PPARγ2, an essential regulator of energy metabolism and insulin sensitivity, stimulates adipocyte and suppresses osteoblast differentiation and bone formation, and correlates with decreased bone mass and increased fracture rate. In contrast, activation of Wnt signaling promotes osteoblast differentiation, augments bone accrual and reduces total body fat. This study examined the cross-talk between PPARγ2 and β-catenin, a key mediator of canonical Wnt signaling, on MSC lineage determination. Rosiglitazone-activated PPARγ2 induced rapid proteolytic degradation of β-catenin, which was prevented by either inhibiting glycogen synthase kinase 3 beta (GSK3β) activity, or blocking pro-adipocytic activity of PPARγ2 using selective antagonist GW9662 or mutation within PPARγ2 protein. Stabilization of β-catenin suppressed PPARγ2 pro-adipocytic but not anti-osteoblastic activity. Moreover, β-catenin stabilization decreased PPARγ2-mediated insulin signaling as measured by insulin receptor and FoxO1 gene expression, and protein levels of phosphorylated Akt (pAkt). Cellular knockdown of β-catenin with siRNA increased expression of adipocyte but did not affect osteoblast gene markers. Interestingly, the expression of Wnt10b was suppressed by anti-osteoblastic, but not by pro-adipocytic activity of PPARγ2. Moreover, β-catenin stabilization in the presence of activated PPARγ2 did not restore Wnt10b expression indicating a dominant role of PPARγ2 in negative regulation of pro-osteoblastic activity of Wnt signaling. In conclusion, β-catenin and PPARγ2 are in cross-talk which results in sequestration of pro-adipocytic and insulin sensitizing activity. The anti-osteoblastic activity of PPARγ2 is independent of this interaction.

## Introduction

Regulation of marrow MSC fate toward adipocyte or osteoblast lineage involves multiple mechanisms including modulation of lineage-specific transcription factors [1]. Such modulation may comprise of direct interactions between transcription factors and their co-modulators, which is often coordinated by changes in the activity of signaling pathways. The example of such interaction includes regulation of Wnt signaling and PPARγ2 activity.

PPARγ nuclear receptor is an essential regulator of energy metabolism and a key transcription factor for adipocyte differentiation [2]. The transcriptional activity of PPARγ is controlled by binding of lipophilic ligands to the ligand binding pocket. The natural ligands consist of polyunsaturated fatty acid derivatives and eicosanoids [2]. Synthetic ligands include a class of antidiabetic drugs, thiazolidinediones (TZDs), which bind to PPARγ with high affinity, activate its adipogenic activity, and act as insulin sensitizers [2]. PPARγ protein is expressed in mice and humans as two different isoforms, PPARγ1 and PPARγ2, due to alternative promoter usage and alternative splicing [3]. In mice, PPARγ2 differs from PPARγ1 by the presence of 30 amino acids (28 amino acids in humans) located at the N-teminus of the AF-1 domain. PPARγ1 is ubiquitously expressed, whereas PPARγ2 expression is restricted to adipocytes, including marrow adipocytes [2], [4]. Although both isoforms have overlapping transcriptional activities, PPARγ2 seems to be more specific for lipids and carbohydrates metabolism. The most common PPARγ polymorphism (Pro12Ala), which is associated with alterations of physiological metabolic status, is located in the unique AF-1 domain of PPARγ2 protein [5], and PPARγ2 but not PPARγ1 can restore adipocytic differentiation in cells previously ablated from both PPARγ isoforms [6], [7]. The studies of the PPARγ role in marrow MSCs differentiation suggest PPARγ2 function in commitment to adipocyte lineage, while PPARγ1 in control of osteoblast differentiation and production of mineralized matrix [4], [8], [9].

PPARγ2 expression and activity increases in marrow MSCs with aging and upon treatment with TZDs, and it correlates with decreased number of osteoblasts and decreased bone formation, and increased number of adipocytes and accumulation of fat in the bone marrow [10], [11]. In contrast, insufficiency in PPARγ activity in MSCs leads to increased number of osteoblasts and increased bone mass, and decreased adipocyte number and fat accumulation in the bone marrow [12], [13]. *In vitro* studies suggest a role for PPARγ2 isoform in commitment of marrow MSCs to adipocytic lineage at the expense of osteoblastic lineage [4], [14]. An analysis of PPARγ2 transcriptome of U-33/γ2 cells, which represent a model of MSC differentiation under the control of PPARγ2, showed that its activation with TZD rosiglitazone (Rosi) leads to simultaneous induction of adipocytic and suppression of osteoblastic gene expression, including suppression of multiple members of Wnt signaling pathway [14]. Although Rosi activates both pro-adipocytic and anti-osteoblastic properties of PPARγ2, these activities can be separated by using ligands of different chemical structures, as we have demonstrated previously [15], [16]. Indeed, the possibility to separate different activities of PPARγ by manipulating with its phosphorylation status has been recently demonstrated in respect to PPARγ anti-diabetic and pro-adipocytic properties. Insulin-sensitizing activity requires blocking phosphorylation of serine 273 [17], while pro-adipocytic activity requires dephosphorylation of serine 112 within PPARγ protein [18], [19]. However, the mechanism by which PPARγ2 acquire anti-osteoblastic activity is not yet elucidated.

**Table 1:** Transcript levels for β-catenin and Wnt10b during treatment with 1 µM Rosi.

Gene	Fold Change (Rosi *vs.*Vehicle)						
	2 h	4 h	6 h	12 h	24 h	48 h	72 h
β-catenin	NC	NC	NC	NC	NC	−2.1	−3.4
Wnt10b	NC	NC	−3.5	−7.4	−52.5	−40.7	−120.1

U-33/γ2 cells were seeded on 6-well plates at the 1×10^4^ cells/cm^2^ density in triplicates and 24 h later media was changed to either 1 µM Rosi supplemented or, as a vehicle, DMSO supplemented. RNA was isolated at the indicated time points from the beginning of treatment. The expression of β-catenin and Wnt10b after Rosi treatment were calculated as fold change as compared to the levels of these transcripts in a parallel culture of vehicle treated cells at the same time points. NC indicates that the tested gene expression in Rosi-treated cells was not significantly different from the expression of this gene in vehicle-treated control. Numbers indicate significant (p<0.05) fold change in transcript levels of tested genes in Rosi-treated *vs.* vehicle-treated cells.

Osteoblast differentiation is regulated by a number of osteogenic pathways, including Wnt signaling [20]. Binding of Wnt glycoprotein ligands to LDL-related protein 5/6 (Lrp5/6) and Frizzled (Fzd) co-receptors triggers release of β-catenin from protein degradation complex, its translocation to the nucleus and activation of TCF/LEF transcriptional complex, which facilitates the expression of canonical Wnt-controlled genes regulating cell proliferation and differentiation [21]. The association between naturally occurring mutations in human Lrp5 receptors and high or low bone mass phenotype demonstrates an essential role of Wnt signaling in the regulation of the skeletal homeostasis [20], [22], [23]. However, the phenotypes of mice with genetically altered components of canonical signaling, including β-catenin and Wnt10b, point toward an interesting phenomenon that different members of the canonical Wnt pathway have distinct effects on the skeleton [24]–[29]. Moreover, they indicate that the same protein may have different functions during MSC differentiation. For example, β-catenin ablation in early mesenchymal progenitors has deleterious effects on skeletal development due to suppressed osteoblast differentiation [25], [30], whereas its ablation in lineage committed osteoblasts increases support for osteoclastogenesis without affecting osteoblastic bone formation [24], [28].

β-Catenin-mediated Wnt signaling requires interaction with other transcriptional regulators. Besides TCF/LEF complex, β-catenin may interact with a number of transcription factors and nuclear receptors including PPARγ [31]–[33]. The interaction between both proteins is facilitated through TCF/LEF binding domain of β-catenin and helices 7 and 8 of PPARγ [33], [34]. It has been demonstrated that pro-adipocytic activity of PPARγ leads to β-catenin dissociation from the complex and its subsequent degradation [33].

Differentiation of marrow MSCs towards osteoblasts relies on functional Wnt10b/β-catenin canonical signaling [35], [36], while their differentiation to adipocytes requires PPARγ2 [4], [12]. Wnt10b/β-catenin signaling suppresses PPARγ2 activity and adipogenesis, while PPARγ2 suppresses Wnt10b/β-catenin signaling and osteoblastogenesis, suggesting fully reciprocal communication between PPARγ2 and canonical Wnt signaling [15], [35]. However, selective activation of PPARγ2 anti-osteoblastic properties leads to suppression of Wnt10b expression, while selective activation of PPARγ2 pro-adipocytic properties does not affect Wnt10b expression [15]. This suggests a possibility that the cross talk between these two regulatory pathways may not be fully reciprocal and may rely in part on a different mechanism, which can be manipulated to advantage one of the MSC phenotypes. Indeed, it has been demonstrated that Wnt signaling, independently of β-catenin activity, may silence PPARγ expression by phosphorylation of histone lysine methyltransferase and recruitment the corepressor complex to PPARγ promoter region [37], [38].

In this study, we demonstrate that PPARγ2 pro-adipocytic and pro-insulin signaling activities require degradation of β-catenin protein and that the degradation of β-catenin does not directly affect anti-osteoblastic activity of PPARγ2. This activity rather depends on Wnt10b, which is under negative control of PPARγ2 and under positive, PPARγ2-independent, control of β-catenin.

**Figure 1:**
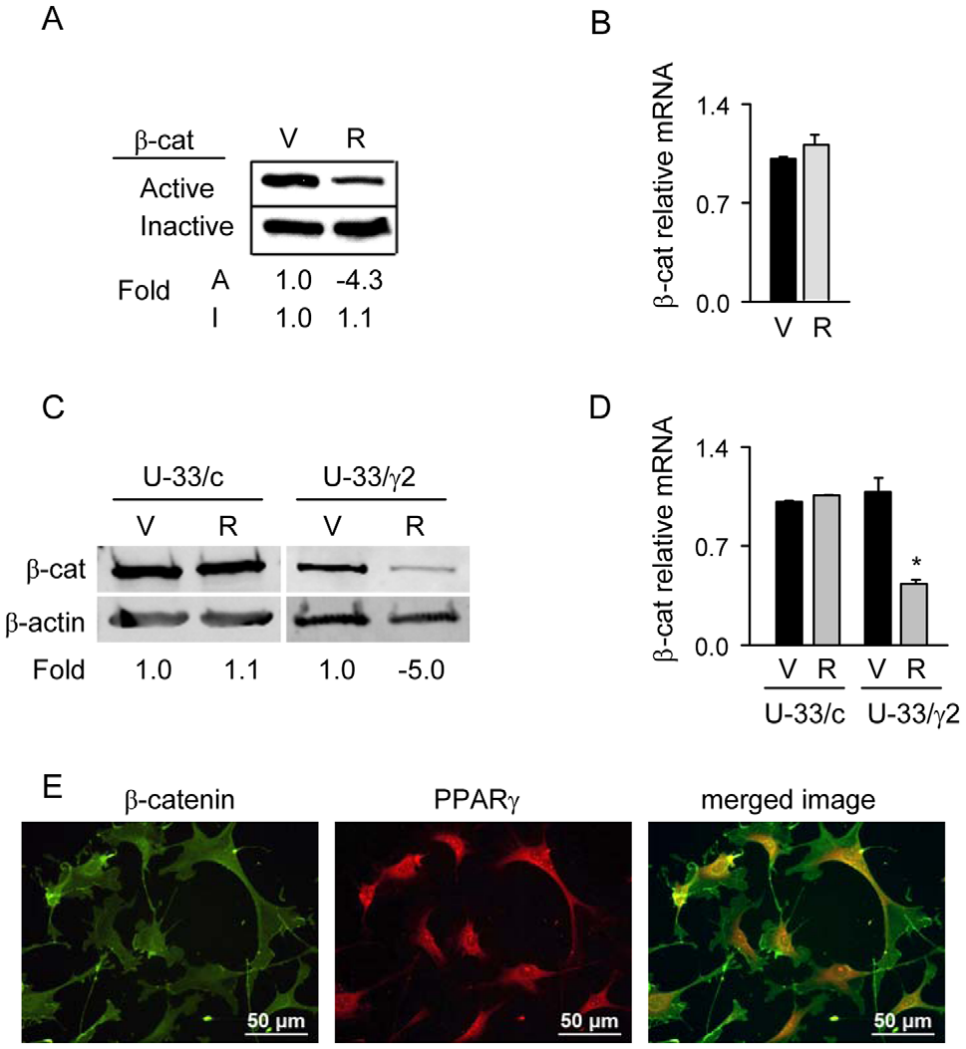
Rosi-mediated activation of PPARγ2 degrades the pool of active, protein-unbound β-catenin. A. Western blot analysis of protein-unbound (Active) and protein-bound (Inactive) fractions of β-catenin isolated from U-33/γ2 cells treated with either vehicle (DMSO) or 1 µM Rosi for 1 h. Protein loading per lane: 3 µg of protein-bound and 50 µg of protein-unbound fraction. B. Relative expression of β-catenin mRNA analyzed after 1 h treatment of U-33/γ2 cells with either vehicle or 1 µM Rosi. C. Western blot analysis of total β-catenin protein levels isolated from U-33/c cells and U-33/γ2 cells treated with either vehicle or 1 µM Rosi for 72 h. Each lane was loaded with 50 µg of total protein lysate. D. Relative expression of β-catenin mRNA analyzed in U-33/c and U-33/γ2 cells after 72 h treatment with either vehicle or 1 µM Rosi. Gene expression is presented as fold difference as compared to levels of β-catenin transcript in vehicle treated U-33/c cells (* p<0.05). E. Immunofluorescent visualization of β-catenin and PPARγ2 proteins in untreated U-33/γ2. V – vehicle; R – Rosi; A – active; I – inactive.

**Figure 2:**
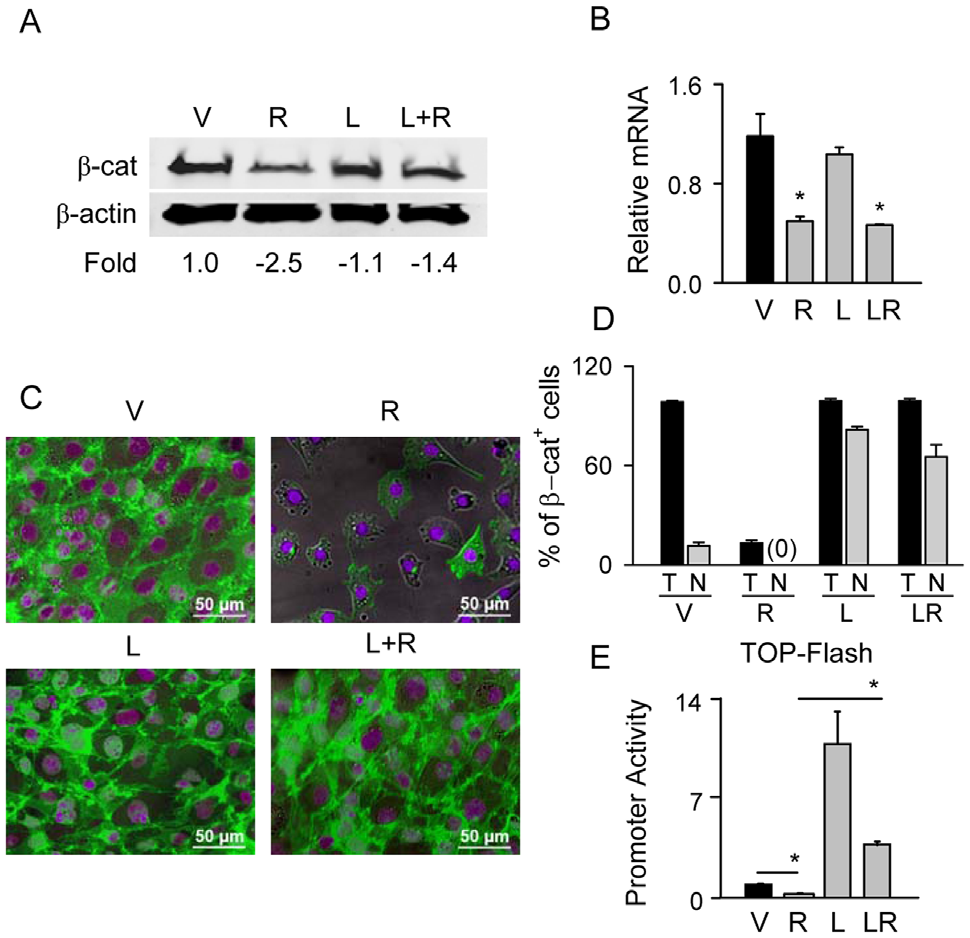
GSK3β antagonist LiCl protects β-catenin protein from PPARγ2-mediated degradation and preserves β-catenin transcriptional activity. U-33/γ2 cells were treated with either vehicle, 1 µM Rosi, 10 mM LiCl, or in combination for 72 h. A. Western blot analysis of total levels of β-catenin protein. β-actin was used as a loading control. Each lane was loaded with 50 µg of total protein lysate. B. Relative expression of β-catenin mRNA as compared to vehicle treated cells. C. Immunocytochemistry of β-catenin protein. Green: β-catenin; purple: DAPI staining of nuclei. D. Percentage of β-catenin positive cells (T) and cells positive for β-catenin in the nucleus (N). E. Transcriptional activity of β-catenin measured in U-33/γ2 cells treated as above for 48 h using TOP-Flash construct in luciferase gene reporter assay. Promoter activity of firefly luciferase was normalized to renilla luciferase which was used as a transfection control (* p<0.05). V – vehicle; R – Rosi; L – LiCl; L+R or LR – LiCl+Rosi.

## Materials and Methods

### Reagents and Antibodies

Specific reagents for this study were obtained from the following sources: MEM-α medium (Invitrogen, Carlsbad, CA), DMEM medium and fetal bovine serum (Hyclone, Logan, UT), G418 (Sigma-Aldrich, St. Louis, MO), protease inhibitor (Thermo Scientific, Rockford, Il), phosphatase inhibitor (Roche, Mannheim, Germany), Opti-MEM, Lipofectamine, and Plus Reagent (Invitrogen), rosiglitazone (Tularik, Inc., San Francisco, CA), GW9662 (GlaxoSmithKline, King of Prussia, PA). The following primary antibodies were used: anti-β-catenin (cat.# 610153, BD Biosciences, San Jose, CA), anti-β-actin (cat.# A1978, Sigma-Aldrich, St. Louis, MO), anti-Akt and anti-phospho-Akt (cat.# 9272 and cat.# 9271, Cell Signaling Technology, Beverly, MA) anti-PPARγ (cat.# sc-22020 and sc-7196, Santa Cruz Biotechnology, Santa Cruz, CA). The following secondary antibodies were used: goat anti-mouse IRDye 800CW and donkey anti-goat IRDye 600 (cat.# 926-32210 and cat.# 926-32224, LI-COR Biosciences, Lincoln, NE), chicken anti-goat Alexa Fluor 594 and goat anti-mouse Alexa Fluor 488 (cat.# A21468 and cat.# A11001, Invitrogen), goat serum (Vector Laboratories, Burlingame, CA). β-Catenin-specific siRNA containing a mixture of four different 20–25 nt oligonucleotides was purchased from Santa Cruz Biotechnology. The following kits were used; BCA Protein Assay (Thermo Scientific), RNeasy Mini (Qiagen, Valencia, CA), DNase I (Invitrogen), iScript cDNA synthesis (Biorad, Hercules, CA), QuikChange Site-Directed Mutagenesis (Stratagene, La Jolla, CA), Power SYBR Green PCR Master Mix (Applied Biosystems, Carlsbad, CA), Dual Luciferase Reporter Assay System (Promega, Madison, WI), Cell Titer 96 AQ_ueous_ Non-Radioactive Cell Proliferation Assay (Promega). All other chemicals and reagents were purchased from Sigma-Aldrich.

### Plasmids

Luciferase gene reporter constructs, TOP-Flash and FOP-Flash (Millipore, Billerica, MA), were used to measure β-catenin transcriptional activity. The TOP-Flash construct contains three copies of the TCF/LEF sites, whereas FOP-Flash has mutated copies of TCF/LEF sites and is used as a control for measuring nonspecific activation of the reporter. β-Catenin expression plasmid constructed on a pSPORT6 vector backbone was purchased from Invitrogen. pEF-PPARγ2 expression construct, a kind gift from Dr. J. Gimble (Pennington Institute, LA), was generated by cloning PPARγ2 cDNA into pEF-BOS expression vector downstream of the translation elongation factor 1α (EF-1α) promoter sequence, which permits the levels of ectopically expressed transcript to be within the physiological range (Figure S1A and B) [39]. PPARγ transcriptional activity was measured using p2AOx construct which has inserted two tandem copies of the rat PPRE sequence specific for acyl-CoA promoter region into pCPSluc vector [40]. Site-directed mutagenesis of the D409 residue of the ligand binding domain (LBD) of PPARγ2 was performed using the following primers: forward 5′-GCA CTG GAA TTA GAT GCC AGT GAC TTG G- 3′ and reverse 5′-CCA AGT CAC TGG CAT CTA ATT CCA GTG C-3′. The accuracy of the introduced mutation was confirmed by DNA sequencing.

**Figure 3:**
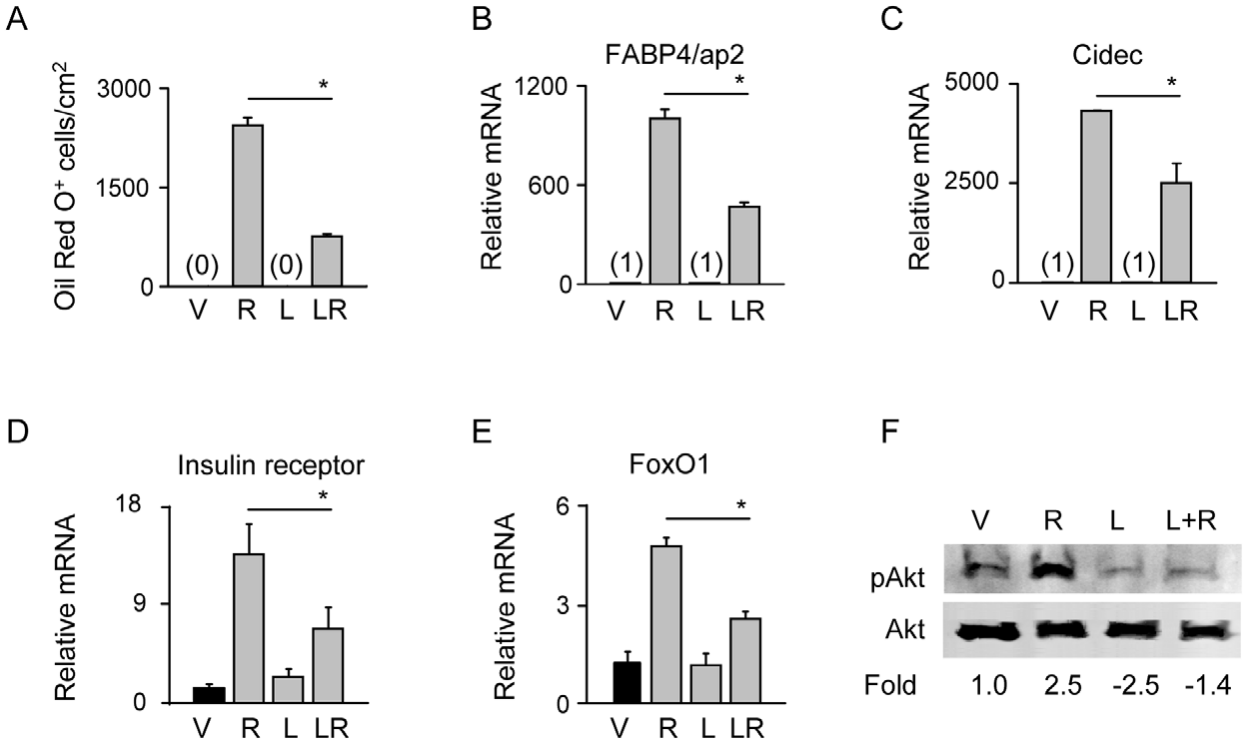
Stabilization of β-catenin suppresses pro-adipocytic activity of PPARγ2 and impairs insulin signaling in U-33/γ2 cells. U-33/γ2 cells were treated for 72 h with either vehicle, 1 µM Rosi, 10 mM LiCl, or in combination. A. Adipocyte differentiation was assessed by measuring the number of Oil Red O positive cells. B–E. Relative expression of adipocyte-specific gene markers (FABP4/aP2 and Cidec) and insulin signaling gene markers (FoxO1 and insulin receptor). Fold change in transcript levels was calculated as compared to vehicle treated cells. F. Western blot analysis of status of Akt posphorylation detected in whole cell lysates using pAkt- and Akt-specific antibodies. Changes in transcript levels and protein levels were calculated as fold as compared to vehicle treated cells. Each lane was loaded with 50 µg of total protein lysate. Statistically significant differences are shown between Rosi-treated samples and samples receiving combined treatment (* p<0.05). V – vehicle; R – Rosi; L – LiCl; L+R or LR – LiCl+Rosi.

**Figure 4:**
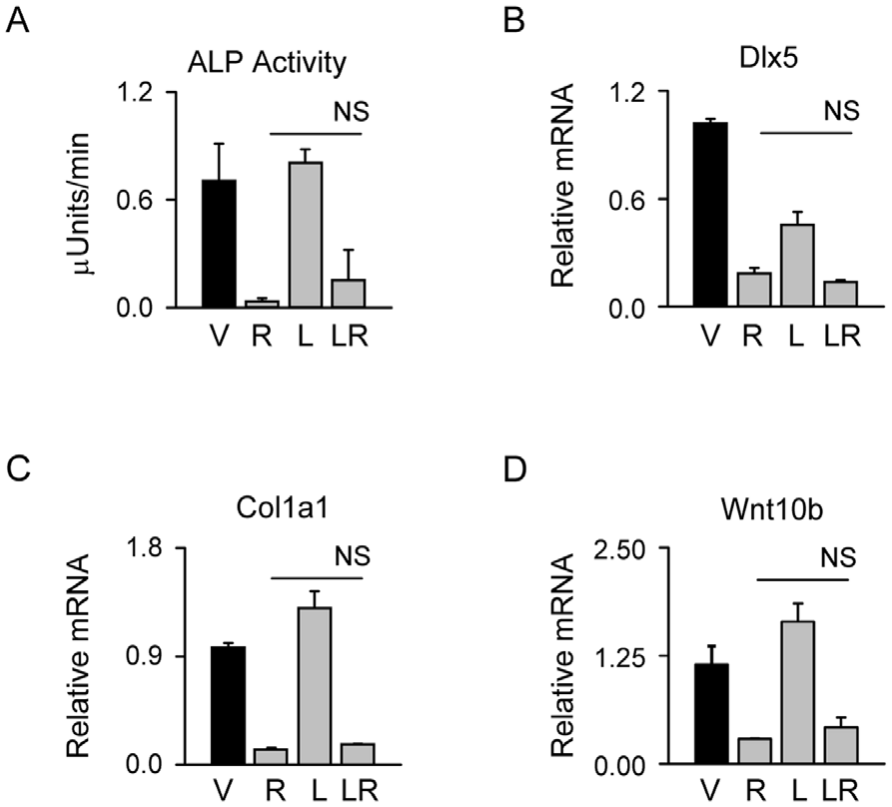
Stabilization of β-catenin protein using LiCl does not affect PPARγ2 anti-osteoblastic activity. U-33/γ2 cells were treated with either vehicle, 1 µM Rosi, 10 mM LiCl, or in combination for 72 h. A. Alkaline phosphatase activity. B–D. Relative expression of osteoblast-specific gene markers and Wnt10b. Fold change in transcript levels was calculated as compared to vehicle treated cells. Statistical differences are shown between Rosi-treated samples and samples receiving combined treatment (NS – non-significant). V – vehicle; R – Rosi; L – LiCl; LR – LiCl+Rosi.

### Cell Lines and Cell Culture Conditions

Murine marrow-derived U-33 cells represent a clonal cell line spontaneously immortalized in the long term bone marrow culture [4]. In basal conditions, the U-33 cells display both pre-osteoblastic phenotype characterized by high alkaline phosphatase enzyme activity and expression of osteoblast-specific gene markers, and preadipocytic phenotype as assessed by levels of expression of adipocyte-specific gene markers [4]. To study the effect of adipocyte-specific transcription factor PPARγ2 on marrow MSC differentiation, U-33 cells were stably transfected with either pEF-PPARγ2 expression construct (referred to as U-33/γ2 cells) or an empty vector pEF-BOS (referred to as U-33/c cells) as described previously [4]. U-33/γ2 and U-33/c cells were maintained in MEM-α supplemented with 10% FBS, 1% penicillin/streptomycin solution (Invitrogen), and 0.5 mg/ml G418 for positive selection of transfected cells. The human kidney HEK293 cells were grown in high glucose DMEM supplemented with 10% FBS and 1% penicillin/streptomycin solution. All cultures were grown at 37C in a humidified atmosphere containing 5% CO_2_.

### Protein Isolation, Fractionation, and Western Blot Analysis

Cell lysate fractionation was performed as described previously [41]. In brief, cells were washed with PBS and scraped into hypotonic lysis buffer (10 mM Tris-HCl pH 7.5, 140 mM NaCl, 5 mM EDTA, 2 mM dithiothreitol, and protease inhibitors), homogenized, and spun at 1,000×g for 10 min at 4C to pellet down nuclei. The remaining supernatant was centrifuged at 100,000×g for 90 min at 4C to yield the high molecular weight protein fraction containing β-catenin bound to the destruction complex (protein-bound or transcriptionally inactive β-catenin) and the cytosol fraction containing β-catenin released from the complex (protein unbound or transcriptionally active β-catenin). For whole cell lysis, cells were scraped into lysis buffer (50 mM Tris-HCl pH 7.5, 150 mM NaCl, 0.5% NP-40, 50 mM NaF, and protease inhibitors) and spun at 7,000×g for 5 min to remove cell debris. For detection of phospho-proteins, cells were scraped into the same lysis buffer and subjected to 5-sec freeze/thaw three times prior to centrifugation at 12,000×g for 5 min. Protein concentration was measured using BCA Protein Assay kit and proteins were separated on 10% SDS-PAGE. For detection of proteins, the following antibodies were used: PPARγ (1∶167), β-catenin (1∶1000), Akt (1∶1000), phospho-Akt (1∶1000), and β-actin (1∶5000). Proteins were visualized using Odyssey Infrared Imaging System (LI-COR Biosciences) after incubation with IR-Dye-conjugated secondary antibodies at dilution of 1∶10,000. A relative quantity of proteins was determined by densitometric measurements of adequate bands using ImageJ (NIH, Bethesda, MD) and values corresponding to fold change in protein expression after normalization to β-actin levels are presented at the bottom of each Western blot image.

### Immunocytochemistry

U-33/γ2 cells were briefly washed with PBS buffer and permeabilized by incubating with ice cold methanol for 10 min. After washing, cells were blocked using 5% goat serum in 0.2% Triton-X for 1 h and incubated with mouse anti-β-catenin (1∶50) and/or goat anti-PPARγ2 (1∶50) diluted in 5% goat serum containing 0.1% Triton-X for 1 h at room temperature. To visualize β-catenin and PPARγ2 cells were incubated with either goat anti-mouse Alexa Fluor 488 or chicken anti-goat Alexa Fluor 594 respectively for 1 h at room temperature. As a negative control, cells were incubated with Alexa Fluor antibodies without prior incubation with primary antibodies. Finally, the cells were mounted using Prolong Gold anti-fade reagent with DAPI (Invitrogen). Images were taken within 24–48 h after immunostaining.

**Figure 5:**
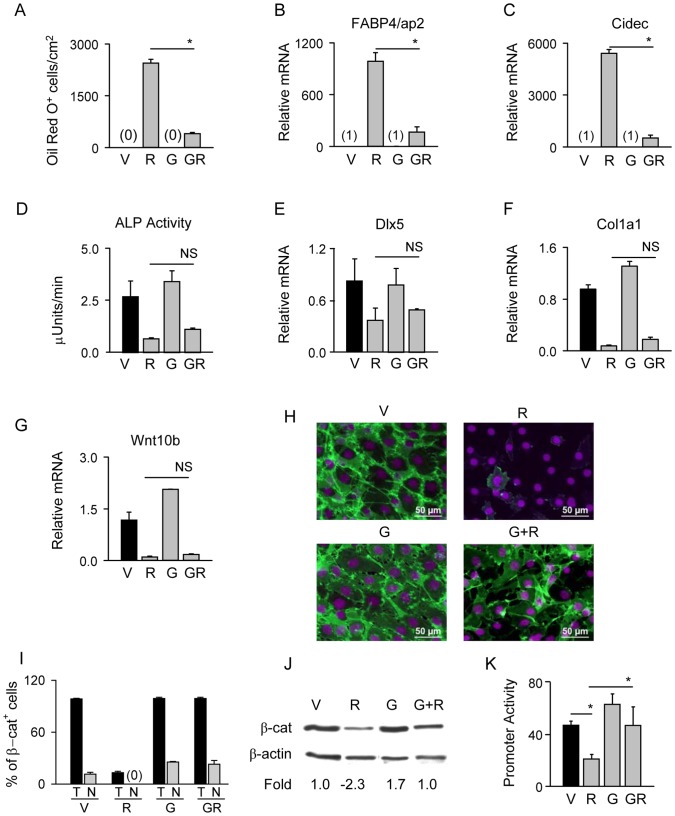
Selective antagonist GW9662 of PPARγ2 pro-adipocytic activity increases β-catenin protein stability. U-33/γ2 cells were treated with either vehicle, 1 µM Rosi, 10 µM GW9662, or in combination for 72 h. A. Adipocyte differentiation was assessed by measuring the number of Oil Red O positive cells. B – C. Relative expression of adipocyte-specific gene markers. D. Osteoblast differentiation was assessed by measuring alkaline phosphatase activity. E – G. Relative expression of osteoblast-specific gene markers and Wnt10b. Fold change in transcript levels was calculated as compared to vehicle treated cells. H. Immunocytochemistry of β-catenin protein. Green: β-catenin; purple: DAPI staining of nuclei. I. Percentage of β-catenin positive cells (T) and cells positive for β-catenin in the nucleus (N). J. Western blot analysis of total β-catenin protein levels. Each lane was loaded with 50 µg of total protein lysate. K. Transcriptional activity of β-catenin measured with luciferase gene reporter assay using TOP-Flash construct. Promoter activity of firefly luciferase was normalized to renilla luciferase which was used as a transfection control. Statistically significant differences are shown between Rosi-treated samples and samples receiving combined treatment (* p<0.05; NS – non-significant). V – vehicle; R – Rosi; G – GW9662; GR – GW9662+ Rosi.

**Figure 6:**
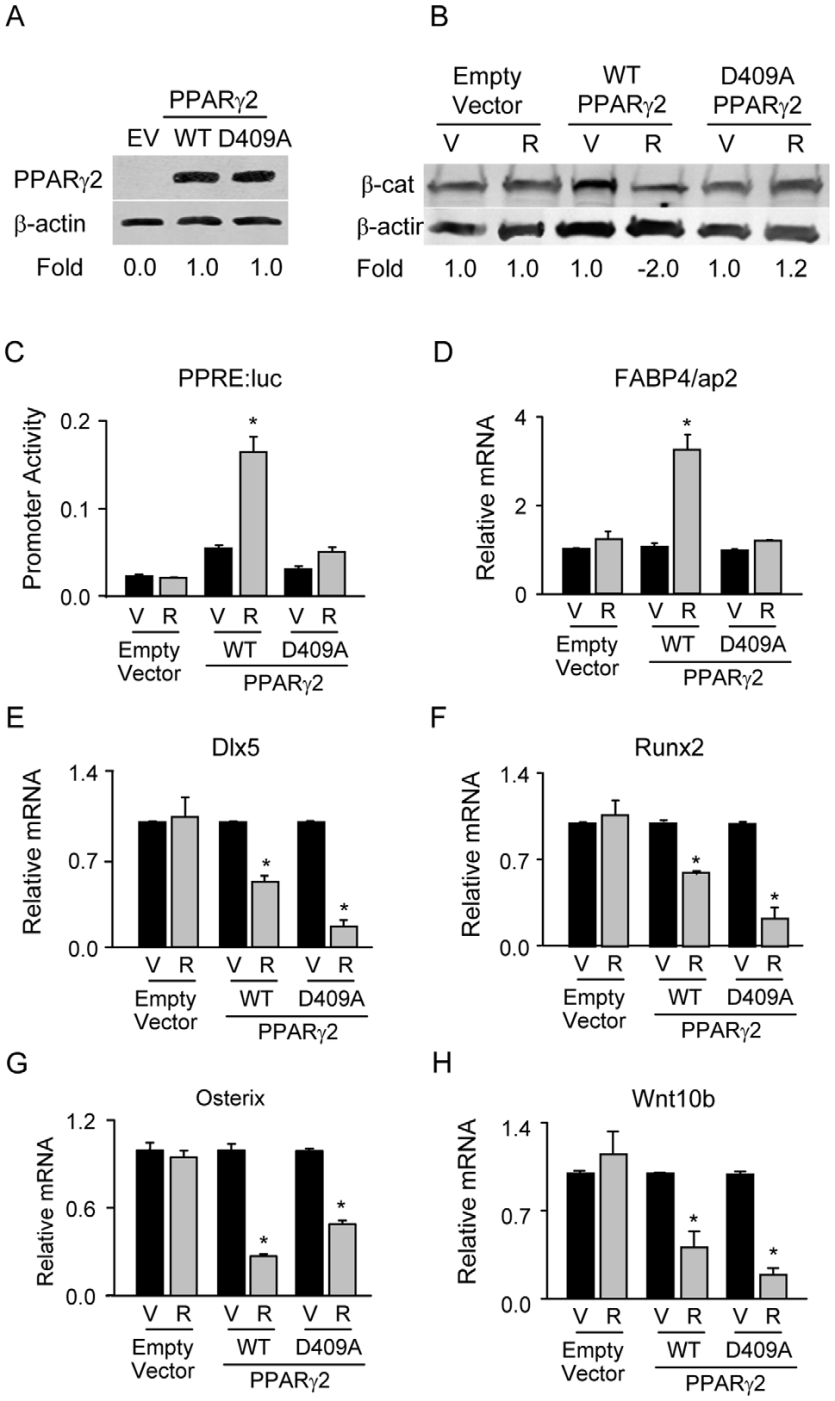
PPARγ2 mutation, abrogating the pro-adipocytic but not the anti-osteoblastic activity, protects β-catenin protein from degradation. A. Western blot analysis of protein levels of non-mutated (WT) and mutated (D409A) forms of PPARγ2 analyzed 72 h after transfection of HEK293 cells. β-actin was used as a loading control. Each lane was loaded with 50 µg of total protein lysate. EV – empty vector control. B. Western blot analysis of β-catenin protein levels after treatment with 1 µM Rosi for 72 h. Hek293 cells were transfected with β-catenin expression construct and either empty expression vectors (pSPORT6 and pEF-BOS), or non-mutated (WT), or mutated (D409A) PPARγ2 expression constructs. Each lane was loaded with 50 µg of total protein lysate. C. Effect of D409A mutation on transcriptional activity of PPARγ2. Hek293 cells were transiently transfected with above constructs and co-transfected with p2AOx luciferase reporter gene construct under the control PPARγ response elements. Cells were treated with either vehicle or 1 µM Rosi for 48 h and lysates were analyzed for luciferase activity. Promoter activity of firefly luciferase was normalized to renilla luciferase which was used as a transfection control. D – G. Effect of D409A mutation on expression of adipocyte-specific (D) and osteoblast-specific (E – G) gene markers, and Wnt10b (H). U-33/c cells were transiently transfected with either empty vector (pEF-BOS), or non-mutated (WT), or mutated (D409A) PPARγ2 expression constructs and treated with either vehicle or 1 µM Rosi for 72 h. Relative transcript levels were calculated as fold change as compared to vehicle treated cells in each transfection. V – vehicle; R- Rosi; * p<0.05 V *vs.* R.

**Figure 7:**
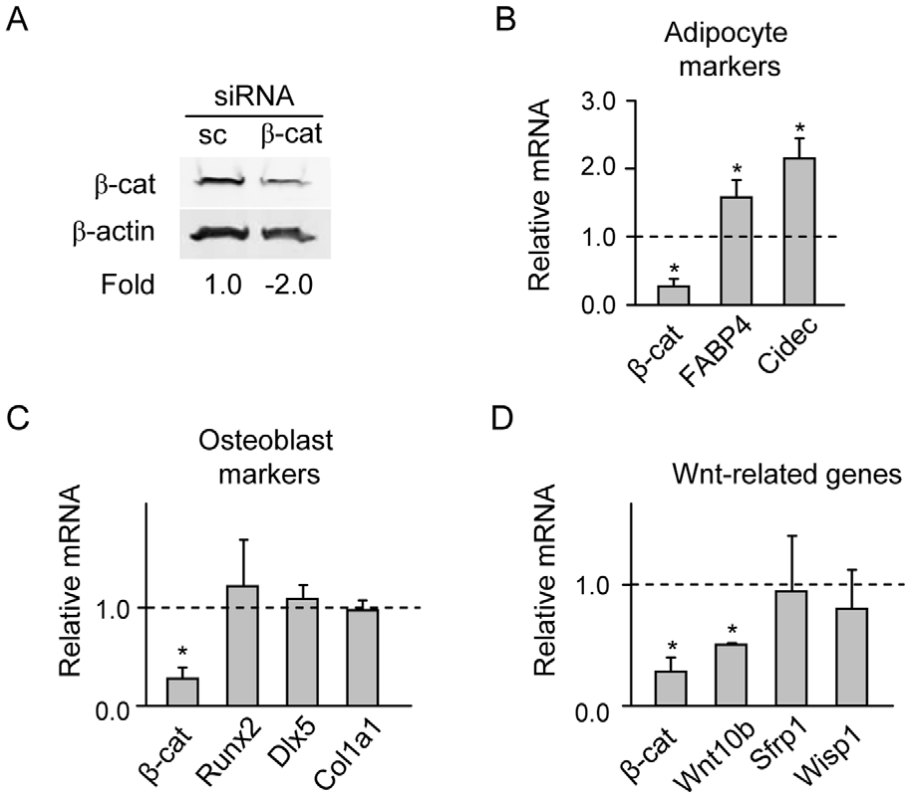
The effect of β-catenin silencing on the expression of adipocyte, osteoblast, and Wnt-signaling gene markers. U-33/γ2 cells were transiently transfected with either 200 ng of β-cat siRNA, which consisted of four β-catenin-specific 20–25 nt oligonucleotides, or 200 ng of scrambled siRNA (sc siRNA) for negative control. Seventy two hours after transfection proteins and RNA were extracted. A. Western blot analysis of β-catenin protein levels. Each lane was loaded with 50 µg of total protein lysate. B–D. Analysis of gene expression of adipocyte-specific (B), osteoblast-specific (C), and Wnt signaling (D) gene markers. All values are expressed as fold change compared to control transfected with sc siRNA and represented by value 1. * p<0.05.

### Quantitative Real-time RT-PCR Analysis

Total RNA was extracted using RNeasy Mini kit. Its purity and concentration were determined using Agilent 2100 Bioanalyzer (Agilent Technologies, Santa Clara, CA). After DNase treatment, 0.75µg of RNA was converted to cDNA using the iScript cDNA synthesis kit. The amount of cDNA corresponding to 7.5 ng of RNA was used for each reaction containing Power SYBR Green mix and was processed using StepOne Plus System (Applied Biosystems, Carlsbad, CA). Relative gene expression was determined by the ΔΔ-Ct method using 18S RNA levels for normalization. Primers were designed using Primer Express 3.0 software (Applied Biosystems). All primers used in this study are listed in Table S1.

### β-Catenin Knockdown using siRNA

U-33/γ2 cells (1×10^5^ cells) were transfected with 200 ng of β-catenin siRNA or nonspecific random siRNA as a negative control using Lipofectamine 2000. Seventy two hours after transfection, total RNA and protein were extracted and analyzed for β-catenin knockdown and expression of target genes.

### Luciferase Gene Reporter Assay

β-Catenin transcriptional activity was measured in U-33/γ2 cells. Cells were seeded in a 24-well plate at the 1×10^4^ cells/cm^2^ density and transfected with 0.3 µg of either TOP-Flash or FOP-Flash plasmid. Twenty four hours later, cells were treated with either 1 µM Rosi, 10 mM LiCl, 10 µM GW9662, or a combination of Rosi with either LiCl or GW9662 for 48 h and luciferase activity was measured using Dual Luciferase Reporter Assay System. Transcriptional activites of PPARγ2 constructs were measured in Hek293 cells. Cells were seeded in a 24-well plate at the 9×10^4^ cells/cm^2^ density and transfected with 0.2 µg of either pEF-BOS empty vector or non-mutated or mutated pEF-PPARγ2 expression plasmids, mixed with 0.2 µg of either pSPORT6 empty vector or pSPORT6 expression plasmid containing wild type β-catenin. All transfection mixtures contained 0.2 µg of p2AOx luciferase reporter construct and 0.02 µg of renilla reporter construct for normalization of transfection efficiency. Twenty four hours after transfection, cells were treated with 1 µM Rosi for the next 24 h and luciferase activity was measured.

### Analysis of Adipocyte and Osteoblast Differentiation of U-33/γ2 cells

For adipogenesis assay, U-33/γ2 cells were seeded in 6-well plate at the 1×10^4^ cells/cm^2^ density. Twenty four hours later, they were treated with either 1 µM Rosi, 10 mM LiCl, 10 µM GW9662 or a combination of Rosi with either LiCl or GW9662 for 3 days. Lipid accumulation was assessed using Oil Red O staining [15]. For analysis of osteoblast differentiation, the U-33/γ2 cells were seeded in 96-well plate at the 1×10^4^ cells/cm^2^ density. After 24 h of growth, they were treated with either 1 µM Rosi, 10 mM LiCl, or 10 µM GW9662 or a combination of Rosi and LiCl or GW9662 for 3 days. Cells were washed with HEPES and alkaline phosphatase (ALP) activity was measured as previously described [15]. The ALP activity was normalized to cell number measured using Cell Titer 96 AQ_ueous_ Non-Radioactive Cell Proliferation Assay kit.

### Statistical Analysis

All experiments were performed in triplicates. Statistical analysis of results was conducted using one-way ANOVA and t-test, as applicable. All data showed represent means and standard deviation of the means (SD). Statistical significance was set to p<0.05.

## Results

### Rosi-activated PPARγ2 Decreases β-catenin Protein Levels

The U-33/γ2 cells, and their negative control U-33/c cells, represent a model of marrow MSC differentiation under control of PPARγ2 transcription factor [4]. Stable transfection with PPARγ2 under the control of elongation factor 1α (EF1α) promoter in U-33/γ2 cells produces basal expression of PPARγ2 transcript (Figure S1A) and protein (Figure S1B). Activation of ectopic PPARγ2, but not naturally expressed PPARγ1, with TZD Rosi converts U-33/γ2 cells to terminally differentiated adipocytes (Figure S1C), while suppressing osteoblast phenotype of U-33 cells and expression of numerous members of the Wnt signaling pathway, including β-catenin [4]; [14]. A detailed analysis of β-catenin gene expression as a function of time following Rosi treatment showed that a decrease in the level of β-catenin transcript occurred relatively late, when cells acquired phenotype of fully differentiated adipocytes marked by significant accumulation of fat droplets and expression of lipid metabolism gene markers (Table 1) [14]. The decrease in β-catenin expression was preceded by a decrease in Wnt10b expression, which occurred as early as 6 h after treatment, the time point which marks in U-33/γ2 cells a state of fate determination (Table 1) [14].

Despite the late response of β-catenin gene expression, its protein levels were decreased much earlier after Rosi treatment (Figure 1). In cytoplasm, a majority of β-catenin protein is sequestered between two different forms, either bound to the multiprotein complex which targets it for proteolytic degradation (protein-bound or inactive form) or free of the complex en route to the nucleus to function as a transcriptional regulator (protein-unbound or active form) [21]. To distinguish between transcriptionally active and inactive forms of cytosolic β-catenin, protein lysates were fractionated as described in Material and Methods to yield protein bound and protein unbound forms of β-catenin, respectively. As shown in Figure 1A, the fraction of protein-unbound β-catenin decreased by 4-fold in U-33/γ2 cells after 1 h treatment with Rosi. No decreases in the level of protein-bound β-catenin and in the level of β-catenin transcript, were observed at this time point (Figure 1A and 1B). After 72 h treatment, the protein level of total β-catenin was decreased by 5-fold (Figure 1C) and was paralleled with a decrease in transcript levels by 2.5 fold (Figure 1D). No change in β-catenin transcript and protein levels were observed at this time point in control U-33/c cells treated with Rosi (Figure 1C and 1D). Interestingly, the basal levels of β-catenin protein in untreated U-33/γ2 cells were lower as compared to untreated U-33/c cells suggesting that even a sole presence of non-activated PPARγ2 isoform has a negative effect on the levels of β-catenin protein._Immunofluorescence analysis of PPARγ2 and β-catenin cellular localization showed that in untreated cells both proteins localize in the cytoplasm, where they may physically interact, as demonstrated previously (Figure 1E) [33].

Presented results indicate that the PPARγ2 negative regulation of β-catenin protein levels involves two mechanisms; a rapid proteolytic degradation and a long-term suppression of β-catenin gene expression.

### Stabilization of β-catenin with LiCl Protects from PPARγ2-mediated Degradation

Phosphorylation by glycogen synthase kinase 3β (GSK3β) targets β-catenin for proteosomal degradation. LiCl prevents β-catenin phosphorylation which includes inactivating autophosphorylation of GSK3β [21]. LiCl treatment of U-33/γ2 cells counteracted the negative effect of Rosi on β-catenin protein levels (Figure 2A and 2C) without counteracting Rosi negative effect on its transcript levels (Figure 2B). Moreover, LiCl treatment resulted significant translocation of β-catenin to the nucleus, which still occurred in cells treated simultaneously with LiCl and Rosi (Figure 2D, Figure S3). This suggests that inhibition of GSK3β activity with LiCl prevents proteolytic degradation of β-catenin and that GSK3β is implicated in β-catenin degradation after Rosi treatment.

Consistent with β-catenin translocation to the nucleus, a protective effect of LiCl was also seen at the level of β-catenin transcriptional activity tested in luciferase gene reporter assay using TOP-Flash construct carrying β-catenin responsive TCF/LEF elements (Figure 2E). As expected, luciferase activity was significantly decreased by Rosi-activated PPARγ2 (by 4-fold), while this activity was increased by over 12-fold in the presence of LiCl. Consistent with β-catenin stabilization and nuclear translocation, simultaneous treatment with Rosi and LiCl not only preserved basal β-catenin activity, but even increased it by 4-fold of that of vehicular control (Figure 2E).

### Stabilization of β-catenin Suppresses PPARγ2 Pro-adipocytic and Insulin Sensitizing Activity, but not Anti-osteoblastic Activity

In U-33/γ2 cells, activation of PPARγ2 with Rosi increases expression of adipocyte-specific genes, induces adipocyte formation, and simultaneously decreases the expression of osteoblast-specific gene markers and suppresses osteoblast phenotype [14]. Stabilization of β-catenin with LiCl in the presence of Rosi-activated PPARγ2 significantly inhibited adipocyte development, as measured by intracellular lipids accumulation (Figure 3A), and suppressed the expression of genes positively regulated by this transcription factor including FABP4/aP2 and Cidec (Figure 3B and 3C). To verify the role of GSK3β in PPARγ2 mediated degradation of β-catenin and suppression of adipogenesis, U-33/γ2 cells were treated with a GSK3β-specific reversible competitive ATP inhibitor 6-6-bromoindirubin-3′-oxime (BIO). As showed in Figure S2A and S2B, BIO treatment offered partial protection of β-catenin in the presence of Rosi and subsequently inhibited adipogenesis as measured by formation of lipid droplets.

Since PPARγ activation with anti-diabetic Rosi increases insulin signaling in adipocytes, we examined the effect of β-catenin stabilization on the expression of gene markers of this pathway. Upon Rosi treatment, both insulin receptor and FoxO1 gene expression increased respectively by 14- and 5-fold, however stabilization of β-catenin in the presence of activated PPARγ2 decreased this effect by 2-fold (Figure 3D and 3E). Furthermore, β-catenin stabilization prevented phosphorylation of Akt, which is a downstream mediator of insulin signaling and indicator of cell sensitivity to insulin (Figure 3F). These results suggest that stabilization of β-catenin suppresses positive adipocytic and insulin sensitizing PPARγ2 activities.

In contrast, β-catenin stabilization did not protect against the PPARγ2-mediated suppression of osteoblast phenotype. As shown in Figure 4A, alkaline phosphatase (ALP) enzyme activity was decreased by Rosi and was not restored in the presence of LiCl. Similarly, LiCl did not protect from PPARγ2 suppressive effects on the expression of Dlx5, Col1a1 and Wnt10b (Figure 4B–D). This indicates that the status of β-catenin protein is in relationship to the positive pro-adipocytic and insulin sensitizing PPARγ2 activities, but not to the suppressive anti-osteoblastic activity.

### Inhibition of PPARγ2 Pro-adipocytic Activity Stabilizes β-catenin and Mimics LiCl Effect

In order to assess a contribution of PPARγ2 pro-adipocytic activity to β-catenin stability, we inhibited Rosi-induced PPARγ2 activity with GW9662 selective antagonist previously shown to block adipogenesis induced by TZD treatment [42]. In U-33/γ2 cells, GW9662 inhibited Rosi-induced lipid accumulation and expression of FABP4/aP2 and Cidec (Figure 5A–C) but it did not affect Rosi-induced suppression of ALP activity and expression of Dlx5, Col1a1 and Wnt10b (Figure 5D–G). Since the pattern of U-33/γ2 cells response to GW9662 was identical to the pattern observed in the presence of LiCl, we analyzed β-catenin protein degradation status. As shown in Figure 5H–J, GW9662 prevented β-catenin protein degradation mediated by Rosi-activated PPARγ2 and restored β-catenin localization in the nucleus (Figure S3). Consistently, GW9662 restored β-catenin transcriptional activity as measured in TOP-Flash gene reporter construct (Figure 5K). Similar to LiCl (Figure 2B), treatment with GW9662 alone did not affect β-catenin transcript levels and treatment in combination with Rosi did not prevent Rosi negative effect on β-catenin transcript levels (data not shown).

To test whether PPARγ2 anti-osteoblastic activity is dependent on the protein domain conferring the pro-adipocytic activity and β-catenin degradation we introduced previously reported mutation of PPARγ1 into PPARγ2 protein sequence [33]. It has been shown that substitution in the PPARγ1 protein sequence of aspartic acid in the position 379 with alanine abrogates proadipocytic activity, prevents β-catenin binding and proteosomal degradation [33]. We introduced the same mutation in the position of D409 of PPARγ2 protein sequence, and verified the stability of D409A mutant in HEK293 cells (Figure 6A), To avoid interference with endogenous non-mutated PPARγ protein, we examined the effect of mutated PPARγ2 on β-catenin stabilization and activity in HEK293 cells, which naturally express minimal amounts of both PPARγ isoforms and β-catenin (data not shown). HEK293 cells were transiently co-transfected with β-catenin and either non-mutated or mutated PPARγ2 expression constructs. As expected, activation with Rosi of non-mutated form of PPARγ2 decreased β-catenin protein levels, however activation of D409A mutant did not have an effect on levels of β-catenin protein (Figure 6B). Consistent with a loss of adipocytic activity, mutation D409A abrogated PPARγ2 transcriptional activity as measured using PPRE-controlled luciferase reporter gene assay (Figure 6C). This result was validated in marrow-derived U-33/c cells. The expression of adipocyte-specific gene marker FABP4/aP2, which is under the control of PPREs, was suppressed in U-33/c cells transfected with D409A construct as compared to non-mutated construct (Figure 6D). Most importantly, mutation D409A retained the suppressive effect of PPARγ2 on the expression of Dlx5, Runx2, and Osterix confirming that the anti-osteoblastic activity of PPARγ2 is independent of pro-adipocytic activity and β-catenin degradation (Figure 6E–G). Moreover, Wnt10b was also downregulated with mutation D409A and in the presence of stabilized β-catenin providing further proof for a PPARγ2-mediated suppression of Wnt10b independent of β-catenin protein status (Figure 6H). These results together indicate that PPARγ2 pro-adipocytic, but not its anti-osteoblastic acitivity, is responsible for a decrease in β-catenin protein levels and that the anti-osteoblastic activity is independent of PPARγ2 pro-adipocytic activity and interaction with β-catenin.

### siRNAs Silencing of β-catenin Affects Adipocytic but not Osteoblastic Gene Expression

To directly test the role of β-catenin in regulation of PPARγ2 pro-adipocytic and anti-osteoblastic activities, we silenced cellular β-catenin using specific siRNA and analyzed alterations in expression of phenotype-specific gene markers. Down-regulation of β-catenin transcript by 70% (Figure 7B–D), paralleled with a 2-fold decrease in β-catenin protein levels (Figure 7A), significantly enhanced transcript levels for adipogenic gene markers FABP4/aP2 and Cidec (Figure 7B). At the same time, transcript levels for osteoblast-specific gene markers Runx2, Dlx5 and Col1a1 were not affected (Figure 7C), confirming our previous observation that the expression of these genes is not directly controlled by β-catenin. Similarly, expression of Sfrp1 and Wisp1, Wnt signaling components shown previously to regulate osteoblast differentiation [43], [44] and being under the control of PPARγ2 [14] remained unchanged. However, the expression of Wnt10b was decreased by 2-fold of its basal levels (Figure 7D). These data support the suppressive effect of β-catenin on adipogenic gene expression and indicate its positive effect on Wnt10b expression. This observation, together with the results presented in Figure 4D and Figure 5G, suggest that Wnt10b is under control of both β-catenin and PPARγ2.

Since mutation D409A had been characterized as unable to degrade β-catenin, one would expect that high levels of β-catenin will have a positive effect on expression of Wnt10b. This expectation was supported by the observation that β-catenin silencing decreased Wnt10b expression independently of PPARγ2 (Figure 7D). However and as shown in Figure 6H, mutant D409A suppressed Wnt10b expression. This suggests that PPARγ2 negative effect on Wnt10b expression is dominant over β-catenin positive effect, at least in this experimental system.

## Discussion

The results presented here demonstrate that PPARγ2 activities positively regulating adipocyte-specific and insulin signaling-specific gene expression are sequestered through interaction with β-catenin, whereas PPARγ2 anti-osteoblastic activity, which requires suppression of osteoblast-specific transcriptome, is independent of this interaction. We have confirmed that β-catenin degradation is an essential step for a direct activation of PPARγ2 pro-adipocytic transcriptional activity mediated through PPRE [33], [34] and we have shown that β-catenin degradation is also required for induction of mechanisms increasing insulin sensitivity. Most importantly, we have demonstrated that the PPARγ2 anti-osteoblastic activity is regulated by a different mechanism, which does not depend on direct cross-talk with β-catenin but involves negative regulation of Wnt10b expression.

The functional interaction between β-catenin and PPARγ2 is two-directional. Stabilization of β-catenin by inactivation of degradation process with either LiCl or BIO GSK3β inhibitor suppresses pro-adipocytic activity of PPARγ2, whereas inhibition of pro-adipocytic activity of PPARγ2 by either selective antagonist GW9662 or D409A mutation stabilizes β-catenin. At the same time, stabilization of β-catenin in the presence of Rosi does not suppress the PPARγ2 anti-osteoblastic activity.

We hypothesize that PPARγ2 anti-osteoblastic activity results from negative, and β-catenin independent, regulation of Wnt10b expression, which is an essential activator of pro-osteoblastic canonical Wnt signaling. Indeed, Wnt10b pro-osteoblastic and anti-adipogenic activity has been demonstrated in plethora of *in vitro* and *in vivo* studies [26], [35], [36], [45]. Accordingly, overexpression of Wnt10b in MSCs induces osteoblast gene expression and inhibits PPARγ2 expression [35], and ectopic expression of Wnt10b in adipocytes produces animals with high bone mass, which are resistant to the bone loss with aging [26]. In contrast, mice deficient in Wnt10b have low bone mass, affected MSCs proliferation and differentiation, and increased propensity of muscle satellite cells to accumulate fat [36], [46]. We have demonstrated previously that PPARγ2 ligands selective only for pro-adipocytic activity do not affect Wnt10b expression, whereas ligands selective only for anti-osteoblastic activity suppress Wnt10b expression [15]. Here, we have shown that Wnt10b is under the negative control of PPARγ2 anti-osteoblastic activity and this control is independent of β-catenin pool regulating PPARγ2 pro-adipocytic activity.

The possibility to activate β-catenin independently of Wnt signaling has been recently demonstrated in respect to the bone marrow response to mechanical stimuli [47], [48]. It has been shown that under mechanical stress β-catenin suppresses adipocyte differentiation and PPARγ activity through a mechanism which involves inactivation of GSK3β, comprising of mTORC2-mediated phosphorylation of Akt protein and resulting in increased β-catenin stability [47], [48]. Although not investigated here it would be of interest to examine whether the mechanisms of β-catenin destabilization by TZD-activated PPARγ2 employs some of the components which increase its stability and prevent adipogenesis during mechanical stress.

Another important aspect of this study is the regulation of PPARγ insulin sensitizing activity through interaction with β-catenin. The results showed here indicate that degradation of β-catenin positively correlates with increased expression of PPARγ-controlled markers of insulin signaling, including pAkt, whereas stabilization of β-catenin leads to the loss of this positive regulation even in the presence of Rosi. It is well recognized that one of the adverse effects of anti-diabetic TZDs is weight gain due to increased fat mass, which suggests that TZDs anti-diabetic and pro-adipocytic activities are tied. However, as recently reported these two activities are independently linked to the phosphorylation status of two distinct serines within the PPARγ protein [17]–[19]. Although it is highly speculative at this point, our results raise an interesting possibility that β-catenin cross-talk with PPARγ, either through direct interaction or through alteration of GSK3β activity, regulates the phosphorylation of both serine 273 and serine 112, which are essential to the anti-diabetic and the pro-adipocytic activity of this nuclear receptor, and that this interaction is one of the culprits for unwanted effect of TZDs on weight gain.

Currently both clinically approved TZDs, rosiglitazone and pioglitazone, undergo critical evaluation of their clinical use due to adverse cardiovascular, cancer and skeletal effects, nonetheless there is no doubt that PPARγ agonists are the most effective among available anti-diabetic drugs [49]. Therefore, better understanding of mechanisms, which regulate multiple activities of PPARγ nuclear receptor including anti-osteoblastic activity, is important for the development of new class of PPARγ agonists, which will harness selectively the desired insulin sensitizing activity without unwanted effects. Although our studies may not fully reflect functional interaction between PPARγ and β-catenin *in vivo*, because they use a model of U-33/γ2 cells which were specifically designed to study PPARγ2 pro-adipocytic and anti-osteoblastic activities in marrow cells *in vitro*, however they may suggests that in the quest for efficient and safe anti-diabetic PPARγ agonists interaction between β-catenin/PPARγ and Wnt10b/PPARγ should be considered.

## Supporting Information

Figure S1 **Ectopic expression of PPARγ2 under control of elongation factor 1α in U-33/γ2 produces basal expression of PPARγ2 and upon TZD activation commits cells to terminally differentiated adipocytes.** A. Analysis of PPARγ1 and PPARγ2 transcript levels in U-33/c and U-33/γ2 cells. Gene expression is presented as fold difference as compared to PPARγ1 levels in U-33/c cells. B. Western blot analysis of total PPARγ protein levels in U-33/c and U-33/γ2 cells. C. Northern blot analysis of PPARγ target gene FABP4/aP2 upon activation with Rosi indicates that its expression transiently upregulated in U-33/c, whereas its expression is sustained in U-33/γ2 cells (* p<0.05).
(TIF)

Figure S2 **GSK3 β inhibitor 6-bromoindirubin-3′-oxime (BIO) protects β-catenin from PPARγ2 mediated degradation and suppresses adipogenesis.** U-33/γ2 cells were pre-treated with 5 µM BIO for 2 h followed by treatment with either 5 µM BIO or in combination of BIO with 1 µM Rosi for 24 h. Non-treated cells or treated with 1 µM Rosi alone were used as controls. A. Western blot analysis of cytoplasmic levels of β -catenin protein. β -actin was used as a loading control. Each lane was loaded with 30 µg of protein lysate. B. Oil Red O staining of lipids. Red: lipid droplets; Purple: cell cytoplasm. V – vehicle; R – Rosi; B – BIO; BR – BIO+Rosi.
(TIF)

Figure S3 **Representative images of U-33/γ2 cells used for counting populations of β-catenin-positive cells and β-catenin-positive nuclei.** Letter symbols indicate cell treatment; V-vehicle, R-rosiglitazone, LiCl-lithium chloride, LiCl+R-lithium chloride and rosiglitazone, GW-GW9662, GW+R-GW9662 and rosiglitazone. Images are shown in green channels of the RGB images, solid arrows indicate positive nuclei, open arrows indicate negative nuclei. Total number of cells was determined by counting DAPI-stained nuclei on RGB image. Results are presented in graphs on Figure 2D and Figure 5I.
(TIF)

Table S1 **DNA primers used for a determination of gene expression in real time q-PCR.**
(DOC)
