# Supplementary figures and images for: β-Catenin Directly Sequesters Adipocytic and Insulin Sensitizing Activities but Not Osteoblastic Activity of PPARγ2 in Marrow Mesenchymal Stem Cells
Source: PLoS One. 2012 Dec 18;7(12):e51746. doi: 10.1371/journal.pone.0051746 (PMC3525589; doi:10.1371/journal.pone.0051746)

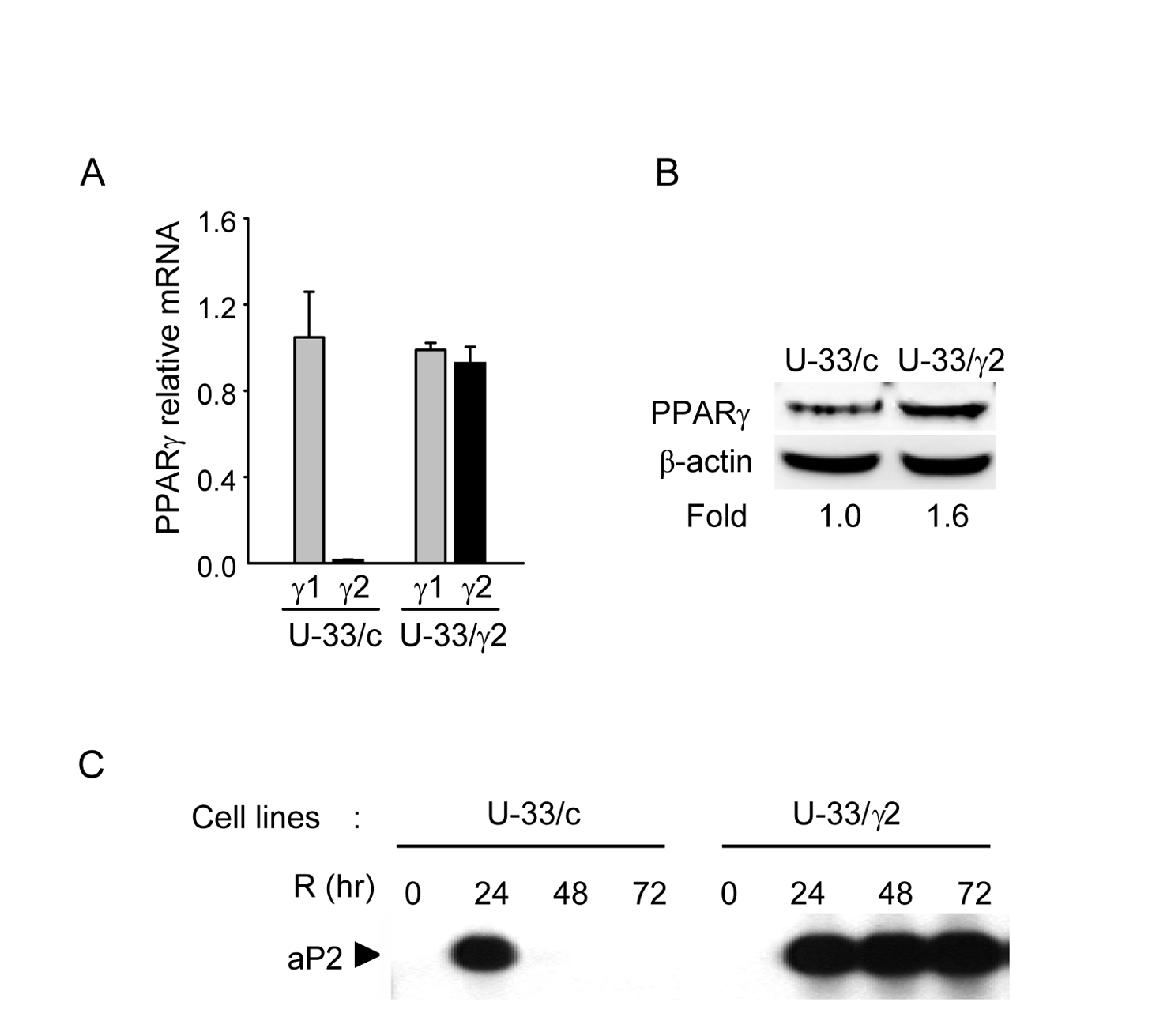

Supplement: Figure S1 — Ectopic expression of PPARγ2 under control of elongation factor 1α in U-33/γ2 produces basal expression of PPARγ2 and upon TZD activation commits cells to terminally differentiated adipocytes. A. Analysis of PPARγ1 and PPARγ2 transcript levels in U-33/c and U-33/γ2 cells. Gene expression is presented as fold difference as compared to PPARγ1 levels in U-33/c cells. B. Western blot analysis of total PPARγ protein levels in U-33/c and U-33/γ2 cells. C. Northern blot analysis of PPARγ target gene FABP4/aP2 upon activation with Rosi indicates that its expression transiently upregulated in U-33/c, whereas its expression is sustained in U-33/γ2 cells (* p<0.05). (TIF) [file pone.0051746.s001.tif]

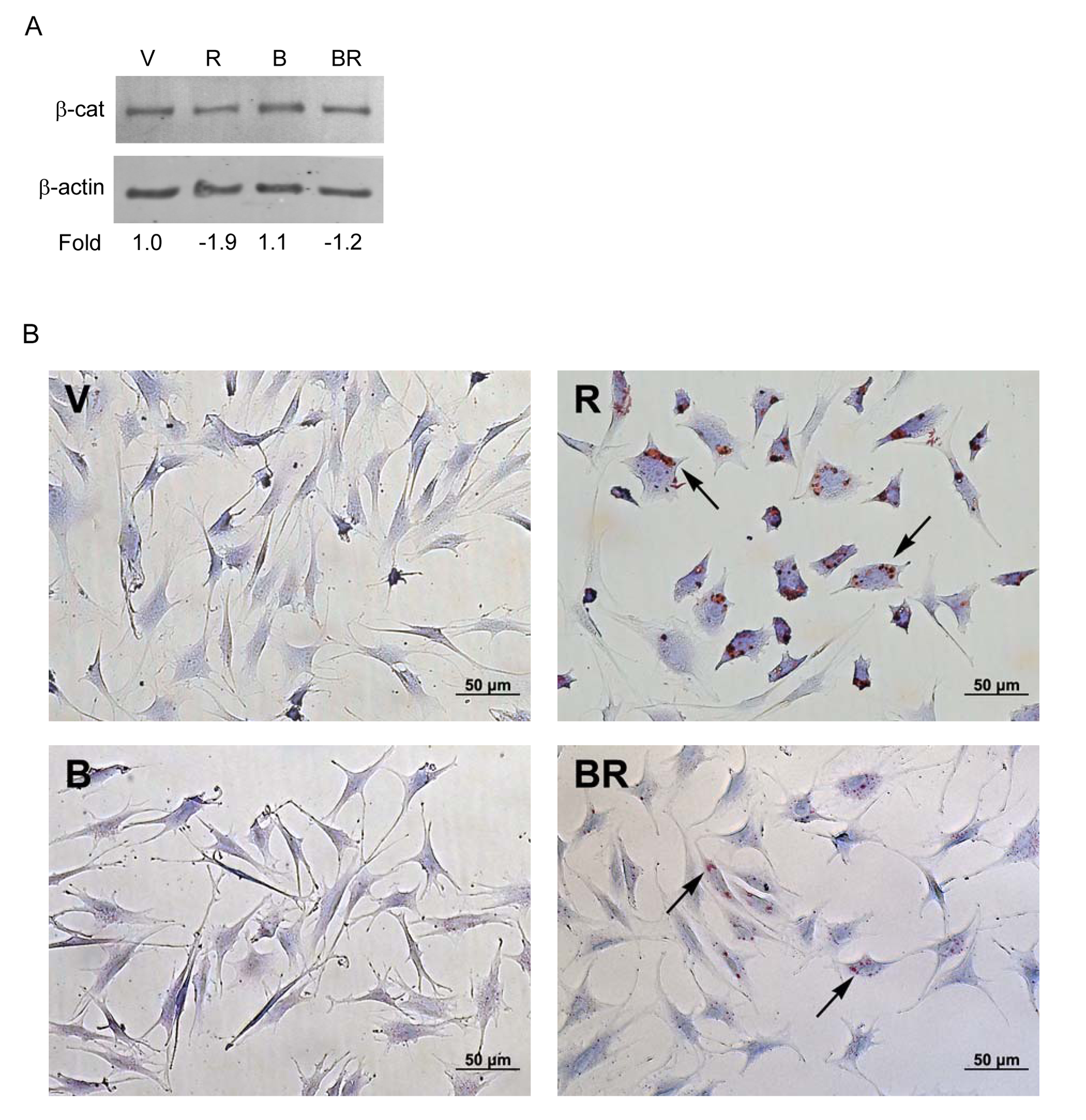

Supplement: Figure S2 — GSK3 β inhibitor 6-bromoindirubin-3′-oxime (BIO) protects β-catenin from PPARγ2 mediated degradation and suppresses adipogenesis. U-33/γ2 cells were pre-treated with 5 µM BIO for 2 h followed by treatment with either 5 µM BIO or in combination of BIO with 1 µM Rosi for 24 h. Non-treated cells or treated with 1 µM Rosi alone were used as controls. A. Western blot analysis of cytoplasmic levels of β -catenin protein. β -actin was used as a loading control. Each lane was loaded with 30 µg of protein lysate. B. Oil Red O staining of lipids. Red: lipid droplets; Purple: cell cytoplasm. V – vehicle; R – Rosi; B – BIO; BR – BIO+Rosi. (TIF) [file pone.0051746.s002.tif]

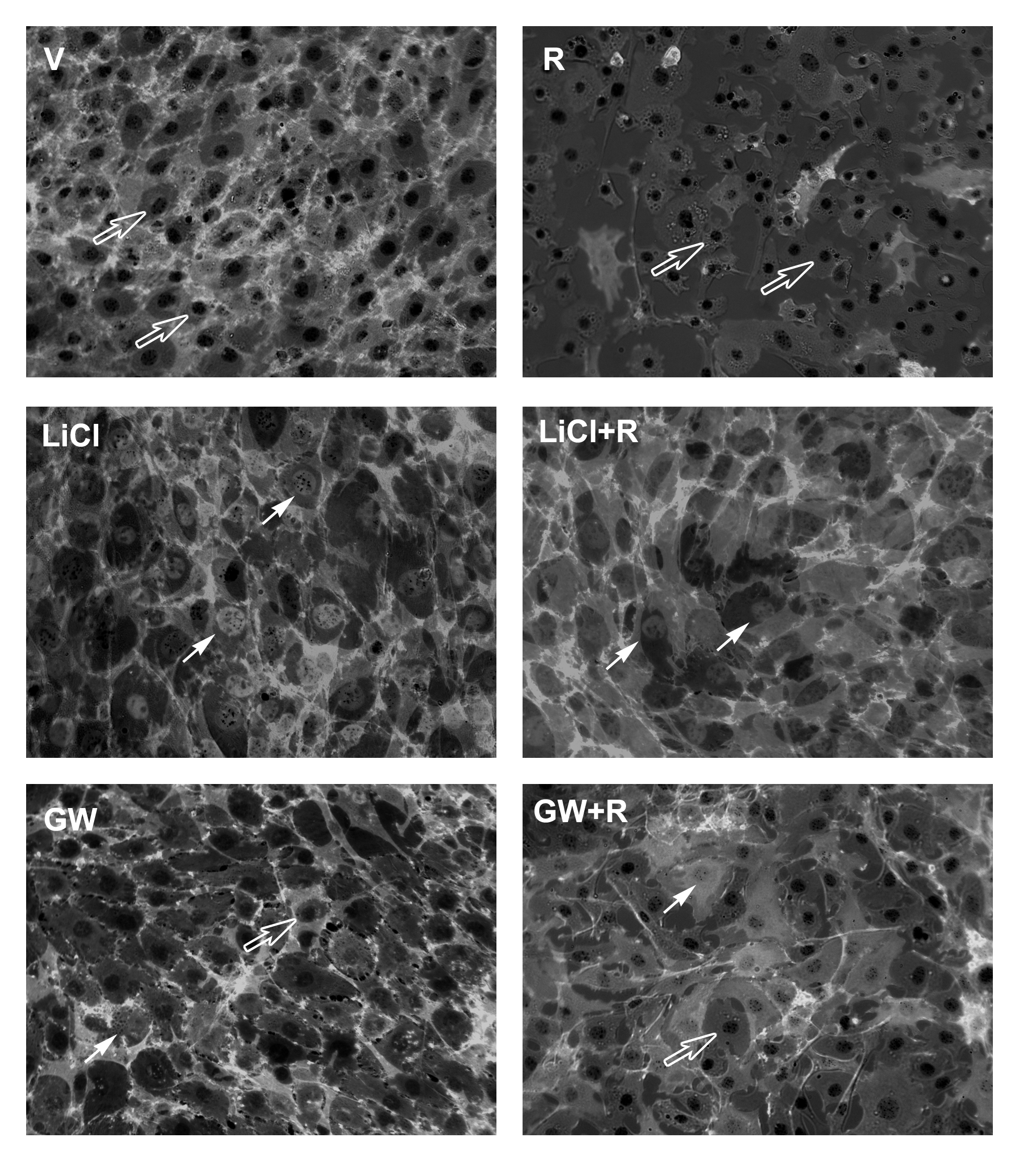

Supplement: Figure S3 — Representative images of U-33/γ2 cells used for counting populations of β-catenin-positive cells and β-catenin-positive nuclei. Letter symbols indicate cell treatment; V-vehicle, R-rosiglitazone, LiCl-lithium chloride, LiCl+R-lithium chloride and rosiglitazone, GW-GW9662, GW+R-GW9662 and rosiglitazone. Images are shown in green channels of the RGB images, solid arrows indicate positive nuclei, open arrows indicate negative nuclei. Total number of cells was determined by counting DAPI-stained nuclei on RGB image. Results are presented in graphs on Figure 2D and Figure 5I. (TIF) [file pone.0051746.s003.tif]
