# Supplementary material for: β-Catenin Directly Sequesters Adipocytic and Insulin Sensitizing Activities but Not Osteoblastic Activity of PPARγ2 in Marrow Mesenchymal Stem Cells
Source: PLoS One. 2012 Dec 18;7(12):e51746. doi: 10.1371/journal.pone.0051746 (PMC3525589; doi:10.1371/journal.pone.0051746)
Supplement: Table S1 — DNA primers used for a determination of gene expression in real time q-PCR. (DOC) [file pone.0051746.s004.doc]

Table S1

| Gene | Forward (5’🡪3’) | Reverse (5’🡪3’) |
| --- | --- | --- |
| Wnt10b | AATGAAGGTGAGCCTCGCC | TGAAAGAGAGCAGCCCTCACA |
| Fabp4 | GCGTGGAATTCGATGAAATCA | CCCGCCATCTAGGGTTATGA |
| Cidec | AGGGAGGGACCTTAGGGAAT | CCAAGTCCAGCTTGGTGAAT |
| Dlx5 | TGACAGGAGTGTTTGACAGAAGAGT | CGGGAACGGAGCTTGGA |
| Col1a1 | ACTGTCCCAACCCCCAAAG | CGTATTCTTCCGGGCAGAAA |
| Insr | GCTGCGTTGTTTACGGGCTGG | GCTCTGGGTGGGTCGCGTTG |
| Foxo1 | CAGTGTGAATCATGGGCAGTT | CGAACATTCTCCCAGAAAAGG |
| Runx2 | GGGCACAAGTTCTATCTGGAAAA | CGGTGTCACTGCGCTGAA |
| Ctnnb1 | TGCGGGAACAGGGTGCTA | TGCGCCGTTGGGTGTC |
| Sfrp1 | GTGGAAGGCAACTCTGTGCA | TGATTCCTGGGCAGCCA |
| Wisp1 | CAGTGACAGCCCTGAATGGTT | CAATCACAGTTTGAAAATAGATAAGTACATACT |
